# Optimizing Scan Range in Computed Tomography of Kidneys, Ureters, and Bladder: A Retrospective Study on Reducing Overscanning

**DOI:** 10.3390/medicina60121952

**Published:** 2024-11-26

**Authors:** Ali Bin Owien, Khaled Alenazi, Ahmad Abanomy, Mansour Almanaa, Mohammad Alarifi, Haitham Alahmad

**Affiliations:** 1 Radiological Sciences Department, College of Applied Medical Sciences, King Saud University, P.O. Box 145111, Riyadh 4545, Saudi Arabia; aliksa87@hotmail.com (A.B.O.); aabanmi@ksu.edu.sa (A.A.); malmanaa@ksu.edu.sa (M.A.); mohalarifi@ksu.edu.sa (M.A.); hnalahmad@ksu.edu.sa (H.A.); 2 Department of Radiology, King Faisal Specialist Hospital and Research Center, P.O. Box. 3354, Riyadh 12713, Saudi Arabia

**Keywords:** overscanning, CT KUB, kidney stone, scan range, KUB

## Abstract

*Background and Objectives*: Computed tomography of the kidneys, ureters, and bladder (CT KUB) is essential for evaluating urinary stones but also exposes patients to significant radiation. The scanning field should be minimized to only the necessary area to limit this radiation exposure. This study aims to assess the extent of CT KUB overscanning in renal colic procedures and identify the appropriate vertebral level for starting CT KUB scans. *Materials and Methods*: A retrospective analysis of 299 adult patients who underwent CT KUB examinations for kidney stone assessment was performed. To assess overscanning, the number of excess slices above the pole of the highest kidney and under the pubic symphysis was measured on the axial image of each patient. To allow for potential human error, a maximum acceptable level of overscanning was set at 10%. *Results*: This study found that only 31% of the scans met the target of less than 10% of overscanning superior to the highest kidney and inferior to the pubic symphysis. In comparison, overscanning was present in 69% of the scans, mainly at a superior level, resulting in higher radiation exposure for patients. *Conclusions*: A significant number of the scans exhibited unnecessary overscanning beyond the highest kidney, prompting us to propose using the upper border of the T10 vertebral body as a potential reference point to establish the upper margin for a CT KUB scan. This study suggests using T10 and the pubic symphysis as reliable landmarks to accurately determine the scan length. Starting CT KUB scans from the T10 vertebral body to the pubic symphysis allows for imaging of the entire urinary tract, minimizing unnecessary overscanning and reducing patient irradiation.

## 1. Introduction

Computed tomography of the kidneys, ureters, and bladder (CT KUB) is the preferred imaging modality for diagnosing suspected urinary tract conditions, particularly kidney stones [1,2]. It provides detailed cross-sectional images of the urinary tract, offering a sensitivity and specificity of 95–100% in detecting even small kidney stones, making it more effective than other imaging techniques, like plain radiography [3,4,5,6,7]. Given its diagnostic advantages, CT KUB remains the first-line imaging technique recommended by both the National Institute of Clinical Excellence (NICE) and the British Association of Urological Surgeons (BAUS) for patients presenting with suspected urinary tract problems [1].

However, a significant drawback of CT imaging is the high radiation exposure it entails compared to other modalities, such as intravenous urography [8]. The radiation dose from standard CT KUB is around 4.5 to 5 mSv, which is greater than from intravenous urography (1.3 to 3.5 mSv) [8]. Exposure to ionizing radiation has been well-documented to increase the risk of developing cancer, particularly when patients undergo multiple scans or when young patients with higher tissue sensitivity are involved [9,10]. Studies have shown that in patients diagnosed with nephrolithiasis, between 17 and 20% were exposed to radiation doses exceeding 50 mSv within the first year of their evaluation and follow-up period, which is well beyond safe limits [11,12]. Multiple factors can increase the likelihood of developing cancer as a result of radiation exposure, including the quantity and duration of radiation exposure, as well as individual characteristics such as age, gender, and tissue sensitivity. Some studies have estimated that the incidence of cancer resulting from a KUB examination ranges from 12 to 35 cases per 100,000 people [13,14]. This raises significant concerns about the cumulative radiation exposure from CT KUB and its potential long-term effects, such as an increased risk of cancer [15]

A key challenge in CT KUB imaging is overscanning, where the scanned area extends beyond the region of interest, contributing to unnecessary radiation exposure. Despite guidelines from the Royal College of Radiologists (RCR), which recommend limiting the scan length to 10% beyond the upper pole of the highest kidney [16], overscanning remains prevalent. Netke et al. (2020) conducted a retrospective study on 100 consecutive patients who underwent CT procedures for renal colic [17]. They examined the number of slices obtained from the CT KUB scan above the highest point of the kidney in relation to scan length. The study found that 81% of scans exceeded the recommended scan length, resulting in higher radiation doses. They reduced the percentage of overscanning to 14% when they optimized the image by confining the scanning body part to the area of interest. By optimizing the scan range to the area of clinical interest, they were able to reduce overscanning to 14% and decrease the dose–length product (DLP) from 319.1 mGy·cm to 280.6 mGy·cm [17]. Similarly, Ghoshal and Giakstas (2021) assessed the extent of unnecessary scanning beyond the uppermost kidney or below the pubic symphysis in each scan and found that this practice was linked to significantly higher radiation exposure [2].

To reduce radiation risks, it is essential to define an optimal starting point for CT KUB scans. Previous studies have suggested that starting the scan at the eleventh thoracic vertebral body (T11) provides comprehensive visualization of the kidneys while limiting unnecessary radiation to surrounding structures [2]. Uldin et al. (2020) found T11 vertebra was shown to be reliable in determining the superior border of the KUB scan, reducing overscanning with no case of underscanning [18]. Therefore, optimizing the scan area based on anatomical landmarks is crucial in reducing patient exposure to radiation while maintaining the diagnostic utility of the scan.

While previous studies have identified overscanning as a significant issue in CT KUB procedures, none have specifically quantified the cancer risk associated with the starting vertebral level of the scan. Our study aims to assess the extent of overscanning and investigate the correlation between different vertebral starting points and the associated radiation dose and cancer risk. This provides a more targeted approach to optimizing CT KUB protocols, offering clearer guidelines for clinical practice. Given the significance of CT KUB scans and the potential risks associated with excessive radiation exposure, this study seeks to evaluate the extent of overscanning and determine the optimal anatomical level for setting the upper boundary of a CT KUB scan. By examining scan data, this study seeks to propose recommendations to minimize radiation exposure while ensuring adequate anatomical coverage.

## 2. Materials and Methods

This retrospective study was performed at a leading hospital in the Riyadh region. Ethical approval was obtained on 17 January 2024 from King Saud University (project number E-23-8350), and a waiver of informed consent was obtained due to its retrospective nature. This study included 299 adult patients (144 females and 155 males) aged between 18 and 93 years, who underwent CT KUB examinations for the assessment of kidney stones. The average age for females and males was 43 ± 16 and 44 ± 17, respectively. The mean Volume CT Dose Index (CTDI_vol_) and DLP for females and males were 8.5 ± 2.7 mGy and 8.0 ± 2.5 mGy, and 390.5 ± 163.7 mGy·cm and 376.7 ± 120.7 mGy·cm, respectively (Table 1). The data, including CT scan images, radiation dose parameters (DLP and CTDI_vol_), and patient demographics, were extracted between January and April 2024 from the picture archiving and communication system (PACS). Patients under 18 or a KUB exam other than colic stones were excluded. CT KUB scans were conducted during full inhalation to optimize the visualization of abdominal organs and reduce motion artifacts.

### 2.1. Overscanning Percentage Calculation

Each scan was viewed using ImageJ software (version 1.54 g, National Institutes of Health, Bethesda, MD, USA) to determine the total number of slices within the scanned range. To assess overscanning, the number of excess slices above the pole of the highest kidney and under the pubic symphysis were counted from the axial stack of images along the longitudinal axis of the scanned patient’s body. Then, the percentage of overscanning was calculated as follows:

$$Overscanning\;Percentage=\left(\frac{Number\;of\;Excess\;slices}{Number\;of\;Slices\;in\;the\;Required\;Scan\;Range}\right)\times 100$$

Compliance with the scanning protocol was then determined by comparing the calculated overscanning percentage to a threshold of 10%, which was set to account for human error [16]. Scans exceeding the 10% overscanning threshold were classified as noncompliant, whereas those at or below this percentage were considered compliant.

**Figure 1:**
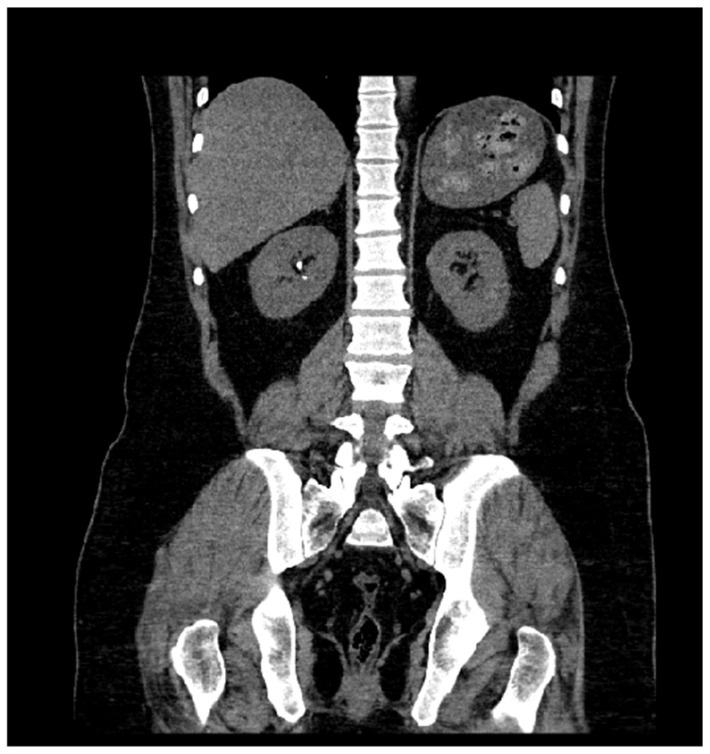
Coronal CT KUB image taken from the PACS database. The right kidney shows several visible hyperdense calcifications, consistent with renal stones. The image also highlights overscanning above the upper poles of the kidneys, which unnecessarily exposes additional tissue to radiation.

In each scan, the starting vertebral level was assessed and compared to the actual vertebral level of the upper pole of the highest kidney. This comparison is to identify the optimal vertebral level at which CT KUB scans should be initiated. The goal is to ensure accurate imaging coverage, minimize unnecessary overscanning, and provide complete anatomical information about the urinary tract (see Figure 1).

### 2.2. Cancer Risk Assessment

To estimate the potential cancer risk associated with CT KUB examinations, this study obtained the DLP data for each patient. The DLP takes into account both the radiation dose and the length of the scanned region. The effective dose (E, mSv) can be estimated by multiplying the DLP by an appropriate conversion factor. The commonly used conversion factor for the abdomen and pelvis region is approximately 0.015 mSv per mGy·cm (for adults) [19]. Then, the estimated potential cancer (R_cancer_) risk can be determined as the product of the effective dose and risk coefficient (5.5 × 10^−2^ Sv^−1^) [20]:

$$R_{cancer}=E\times Risk\;Coefficient$$

### 2.3. Statistical Analysis

The data collected for each patient, including CTDI_vol_, DLP, age, and gender, were analyzed using descriptive statistics. The mean and standard deviation were calculated for each parameter to summarize the radiation dose and demographic information of the studied samples. Microsoft Excel was used to compute these descriptive statistics. 

## 3. Results

As outlined in Table 2, this study analyzed the compliance of the scan extent with the superior (highest kidney) and inferior (pubic symphysis) boundaries. The table summarizes the number of cases that adhered to the scan protocol, particularly regarding the extent of overscanning beyond these anatomical landmarks.

**Figure 2:**
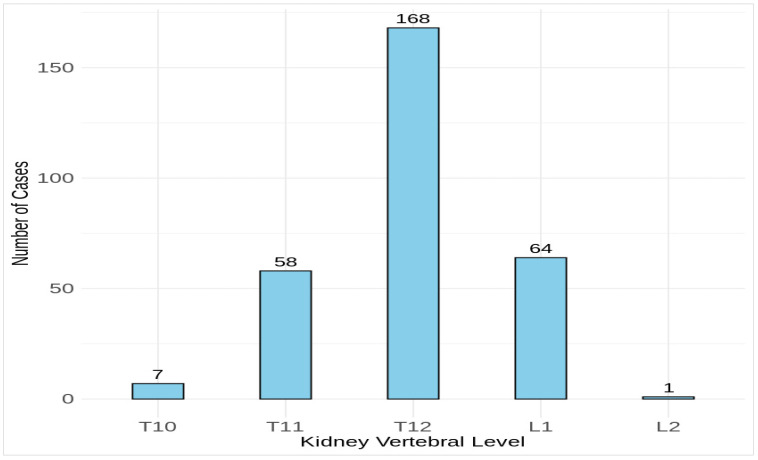
Distribution of the actual vertebral level corresponding to the upper pole of the highest kidney in the studied patients. Vertebral levels are marked from thoracic (T) to lumbar (L).

This study found that 93 cases out of 299 were in overall compliance with both borders and exceeded the highest kidney and pubic symphysis by only 10% or less. However, at a superior extent, 205 cases (68.6%) exceeded the highest kidney by 10.5%–93.0%, and at an inferior extent, only 1 case (0.3%) exceeded the pubic symphysis by 13.4%. There were no scans of noncompliance with both borders. Regarding the kidney vertebral level, Figure 2 shows that all the scanned kidneys were at T10 or below, of which 168 of 299 scans were at T12. The lowest kidney level was at L2 in only one patient. However, this study found that the majority of scan levels were at T10 or above, with approximately 59% of the procedures.

**Figure 3:**
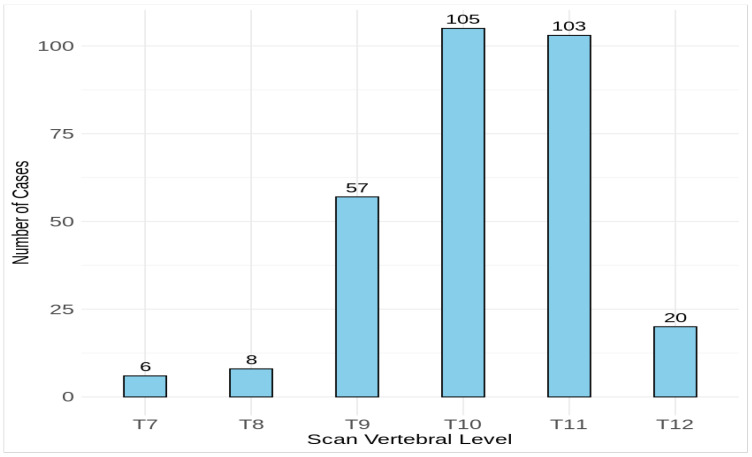
Distribution of the vertebral starting level for CT KUB scans in the studied patients. Vertebral levels are marked from thoracic (T).

The difference between the scan levels and actual levels of the highest kidney suggests that there may be a mismatch between where the scan begins and the real position of the upper pole of the highest kidney, potentially resulting in increased radiation exposure (Figure 3). Scans at the highest vertebral level have the highest mean DLP values. The mean DLP reduced by 45% from 673.4 mGy·cm at T7 to 372.5 mGy·cm at T12. The estimated 45% dose reduction from the optimized protocol represents an average value and does not account for individual variations in CTDI_vol_ due to patient body size. For instance, CTDI_vol_ varied significantly for males (8.5 ± 2.7 mGy), indicating that dose reductions may differ depending on patient-specific factors, such as body size and composition.

Table 3 estimates the mean effective dose and radiation-induced risk for the CT KUB scans at different vertebral levels. This estimation considers various factors, such as the total radiation dose received during the examination and the scan length.

## 4. Discussion

In this retrospective observational study, CT KUB scan data from 299 adult patients were analyzed for kidney stone assessment, aiming to optimize scan protocols and reduce radiation risks. This study assessed overscanning by comparing the number of excess slices above the highest kidney and below the pubic symphysis to the total number of slices, setting an acceptable overscanning limit of 10%. The effective dose and cancer risk associated with different vertebral starting points in CT KUB were identified. This analysis will help minimize unnecessary radiation exposure. 

This study’s findings show that excess overscanning occurred for a high number of patients (n = 206), resulting in a significant increase in radiation exposure. It is important to note that based on the limited information provided by the initial topogram, it can be challenging to precisely locate the kidney and determine the starting point for the scan. Even with a 10% margin of human error, nearly 69% of scans failed to meet this target. One possible reason is that there are other structures, such as the stomach and liver, that overlap. As a result, the operator may set upper scan limits that are higher than required to ensure that the upper part of the higher is included. Furthermore, the operator’s anxiety about overlooking potential abnormalities may contribute to an unnecessary extension of the scan range.

Scan length is a crucial factor in determining the radiation dose for individuals receiving CT scanning. Larger scan lengths give radiation doses to greater portions of the body, increasing patients’ radiation exposure. Radiation exposure can be decreased by confining the scan length to the region of interest, for example, scanning from the top of the kidneys rather than the top of the liver for the examination of kidney stones [21]. Overscanning not only increases radiation exposure for patients but also generates more imaging data that may not significantly contribute to diagnostic accuracy, potentially leading to higher expenses and longer interpretation times. There is a clear trend: as the scan starts at higher vertebral levels, both the effective dose and the associated cancer risk increase. For example, starting at T7 results in an effective dose of 10.1 mSv, almost double the dose at T12, with a cancer risk of 5.6 cases per 10,000 (Table 3). While these values provide a useful comparison, they are based on the current dataset, which inherently includes patient variability. Healthcare practitioners can limit radiation exposure and enhance the overall efficiency of CT KUB scans by employing methods to reduce overscanning, such as precisely establishing scan field boundaries. 

As a general guideline, radiographers attempt to use the diaphragm as a starting point, but this approach sometimes includes an unnecessary portion of the liver and possibly parts of the lung base due to changes in the diaphragm position during inspiration [2]. Uldin et al. (2020) found that the diaphragm is often located a significant distance from the top limit of the kidneys. Even if the diaphragm is closer to the kidneys in the average patient, predicting its position relative to the kidney in a specific patient is challenging. Thus, avoiding overscanning or underscanning the kidney would be challenging [18]. On the contrary, Ghoshal and Gaikstas (2021) found it easy to identify the pubic symphysis as a distinct and visible landmark. This was evident in the significantly lower amount of excessive scanning performed below the symphysis (0.3%).

In order to prevent overscan, Uldin et al. (2020) suggested using an anatomical landmark that is fixed in position relative to the kidneys, close enough to lower scan length, distant enough to prevent underscanning, and easily visible on the coronal scout scan required to calibrate the full CT KUB [18]. The existing literature has identified the utilization of the T11 vertebral level as a reference point in CT scans [2,18]. Although the highest kidney level in 292 out of 299 cases in this study was at T11 or lower, seven cases will not be fully included.

Therefore, this study presents an approach to minimizing overscan in CT KUB investigations using the T10 vertebra as a reliable landmark. This protocol achieves minimal overscan length and prevents underscanning. This study confirms that employing the T10 vertebra significantly reduces radiation exposure to patients. Cavenagh et al. had a similar objective of establishing T10 as the optimal upper limit for the CT KUB test. Their preliminary evaluation revealed that 90% of scans conducted at their facility began at T10 or lower. Based on their analysis, they concluded that scanning all kidneys thoroughly would still be possible if the scans commenced at T11. Consequently, they implemented a new protocol within their department, officially adopting T10 as the upper limit for scans while acknowledging that T11 could also be considered a viable reference point in the future [22]. Maguire et al. found that using T10 as the superior limit of CT KUB lowered patients’ mean dose by 16.2% [23]. Corwin et al. (2014) found that providing the axial scan range from the inferior end plate of the T10 vertebral body to the inferior border of the pubic symphysis completely covers the urinary system, resulting in a 17.7% reduction in scan length compared to the standard abdominopelvic CT methodology [24].

Overall, this study’s results shed light on various aspects of CT KUB scans, including patient demographics, radiation parameters, overscanning patterns, and optimal scan initiation levels. Importantly, the results demonstrate that selecting the optimal scan range can significantly reduce radiation exposure. This reduction in unnecessary exposure minimizes the potential cancer risks (Table 3). These findings can inform recommendations to optimize CT KUB scan protocols, emphasizing the importance of minimizing unnecessary radiation exposure while maintaining diagnostic accuracy. Implementing these recommendations can reduce potential risks associated with CT KUB examinations and promote patient safety and well-being.

This study has a few limitations. Firstly, only adult patients were included. The data used were obtained from a single institution. Patients’ weight, height, and body mass index were not available. The variability in patient body sizes and scan endpoints may affect the accuracy of effective dose and cancer risk estimates, as these estimates rely on average values. Individual variations in body size and scan lengths could lead to disparities in radiation exposure. Therefore, these estimates should be interpreted cautiously. Further studies should consider more consistent patient characteristics and scan parameters to improve the validity of such assessments. Finally, this study did not include long-term follow-up of patients to observe actual cancer incidence. The cancer risk estimates provided are based on theoretical models of radiation exposure.

## 5. Conclusions

In this retrospective study on CT KUB scans for kidney stones, only 31% of the scans showed accurate CT scan extent both superiorly (kidneys) and inferiorly (pubic symphysis), while 69% exhibited overscanning. The effective implementation of a new protocol to reduce radiation exposure during CT KUB exams is discussed. This study proposes using the T10 vertebral body and pubic symphysis as reference points on the coronal scout image to determine the scan length. As a result, the entire urinary tract will be visible. By implementing this method, overscanning will be minimized, effectively reducing the exposure of more tissue to ionizing radiation. Reducing radiation exposure is crucial since cumulative exposure from CT scans may raise the risk of radiation-induced cancers. Thus, implementing this optimized scanning protocol could enhance image quality and diagnostic accuracy while lowering the long-term cancer risk linked to CT KUB exams. Continuous education is necessary to integrate the technique into the usual workflow of gathering CT KUB studies and raising awareness about radiation safety. This study provides valuable insights into the potential cancer risks linked to CT KUB examinations. However, additional long-term research is needed to confirm these results and evaluate the actual cancer risks over longer durations. Such studies would significantly enhance our understanding of the long-term effects of radiation exposure from these procedures.

## Figures and Tables

**Table 1:** Descriptive analysis of the scanning parameters and the resulting radiation dose during CT KUB scanning of the studied sample.

Gender	No. of Exam	Age	Tube Potential (kVp)	Tube Current (mA)	Slice Thickness (mm)	CTDI_vol_ (mGy)	DLP (mGy·cm)
Female	144	43 ± 16	117.3 ± 11.1	620.1 ± 94.7	1.25	8.5 ± 2.7	390.5 ± 163.7
Male	155	44 ± 17	116.7 ± 12.1	628.4 ± 90.7	1.25	8.0 ± 2.5	376.7 ± 120.7

CTDI_vol_: Volume CT Dose Index. DLP: Dose-Length Product.

**Table 2:** Compliance with recommended scan boundaries for CT KUB scans at upper (kidney) and lower (pubic symphysis) borders.

	No. of Exams	Percentage %
Compliance with both borders	93	31.1
Noncompliance with both borders	0	0
Noncompliance with one border:		
• Superior	205	68.6
• Inferior	1	0.3
Total	299	100

**Table 3:** The average effective dose (E) and cancer risk estimate depending on the starting point of the CT KUB scans for the given sample.

CT Vertebral Level	*E* (mSv)	Cancer Risk (Cases per 10,000)
T12	5.6	3.1
T11	5.2	2.8
T10	5.7	3.1
T9	6.4	3.5
T8	6.9	3.8
T7	10.1	5.6

## Data Availability

The data are available upon request from the corresponding author.
